# Interprofessional collaboration in primary health care from the perspective of implementation science

**DOI:** 10.1590/0102-311XEN213322

**Published:** 2023-11-13

**Authors:** Natália de Paula Kanno, Marina Peduzzi, Ana Claudia Camargo Gonçalves Germani, Patrícia Coelho De Soárez, Andréa Tenório Correia da Silva

**Affiliations:** 1 Prefeitura do Município de São Bernardo do Campo, São Bernardo do Campo, Brasil.; 2 Faculdade de Medicina, Universidade de São Paulo, São Paulo, Brasil.; 3 Escola de Enfermagem, Universidade de São Paulo, São Paulo, Brasil.; 4 Faculdade de Medicina de Jundiaí, Jundiaí, Brasil.; 5 Faculdade de Medicina Santa Marcelina, São Paulo, Brasil.

**Keywords:** Implementation Science, Primary Health Care, Patiente Care Team, Health Sciences, Technology and Innovation Management, Ciência da Implementação, Atenção Primária à Saúde, Equipe de Assistência ao Paciente, Gestão de Ciência, Tecnologia e Inovação em Saúde, Ciencia de la Implementación, Atención Primaria de Salud, Grupo de Atención al Paciente, Gestión de Ciencia, Tecnología e Innovación en Salud

## Abstract

The objective was to analyze the perceptions of primary health care (PHC) workers about interprofessional collaboration from the perspective of implementation science. This is a qualitative study that used in-depth interview as a data production technique. Interviews were conducted with 15 workers (three community health agents, one nursing assistant, three nurses, three managers, three physicians, and two nursing technicians) from basic health units in the Municipality of São Bernardo do Campo, São Paulo State, Brazil. The interview plan was based on three domains of the Consolidated Framework for Implementation Research (CFIR). Thematic content analysis was used. In the interprofessional collaboration characteristics domain, respondents highlighted the complexity, and its possible influence, as to the implementation and sustainability of this practice. In the inner setting domain, factors that influence interprofessional collaboration were identified, namely: how the time allocated to formal communication/team meetings is used; social interactions between professionals; and leadership characteristics, such as feedback, autonomy and participation in decisions. In the individuals characteristics domain, participants noted interprofessional collaboration geared to quality of care and the need for integration between knowledge centers. Thus, measures to enhance the quality of communication, collective team building and leadership can contribute to improve interprofessional collaboration in PHC and leverage its impacts on health care.

## Abstract (pt)

O objetivo foi analisar as percepções de trabalhadores da atenção primária à saúde (APS) sobre a colaboração interprofissional na perspectiva da ciência de implementação. Trata-se de estudo qualitativo que utilizou a entrevista em profundidade como técnica de produção de dados. Foram entrevistados 15 trabalhadores (três agentes comunitários de saúde, um auxiliar de enfermagem, três enfermeiros, três gerentes, três médicos e dois técnicos de enfermagem) de unidades básicas de saúde no Município de São Bernardo do Campo, São Paulo, Brasil. O roteiro da entrevista baseou-se em três dimensões do Quadro Conceitual Consolidado para Pesquisa de Implementação (CFIR; *Consolidated Framework for Implementation Research*). Foi realizada análise de conteúdo temática. Na dimensão características da colaboração interprofissional, os entrevistados destacaram a complexidade, e sua possível influência, na implementação e sustentabilidade dessa prática. Na dimensão cenário interno, foram identificados fatores que influenciam a colaboração interprofissional: como se utiliza o tempo destinado a comunicação formal/reuniões de equipe; interações sociais entre os profissionais; e características da liderança, como *feedback*, autonomia e participação nas decisões. Na dimensão características dos indivíduos, os participantes destacaram a colaboração interprofissional direcionada para a qualidade do cuidado e a necessidade de integração entre os núcleos de saberes. Assim, ações para aperfeiçoar a qualidade da comunicação, a construção coletiva em equipe e o aprimoramento da liderança podem contribuir para melhorar a colaboração interprofissional na APS e potencializar seus impactos na atenção à saúde.

## Abstract (es)

El objetivo fue analizar las percepciones de los trabajadores de atención primaria de salud (APS) sobre la colaboración interprofesional desde la perspectiva de la ciencia de implementación. Se trata de un estudio cualitativo que utilizó la entrevista en profundidad como técnica de producción de datos. Fueron entrevistados 15 trabajadores (tres agentes comunitarios de salud, un auxiliar de enfermería, tres enfermeros, tres gerentes, tres médicos y dos técnicos de enfermería) de unidades básicas de salud en el Municipio de São Bernardo do Campo, São Paulo, Brasil. La guía de la entrevista se basó en tres dimensiones del Marco Consolidado para la Investigación sobre la Implementación (CFIR; *Consolidated Framework for Implementation Research*). Se realizó un análisis de contenido temático. En la dimensión característica de la colaboración interprofesional, los entrevistados destacaron la complejidad y la posible influencia en su implantación y sostenibilidad. En la dimensión escenario interno, fueron identificados factores que influencian en la colaboración interprofesional: cómo se utiliza el tiempo destinado a la comunicación formal/reuniones de equipo; las interacciones sociales entre los profesionales, y las características del liderazgo, como *feedback*, la autonomía y la participación en las decisiones. En la dimensión de las características de los individuos, los participantes destacaron la colaboración interprofesional orientada a la calidad de la atención y la necesidad de integración entre los núcleos de saberes. Así, las acciones para mejorar la calidad de la comunicación, la construcción colectiva en equipo y la mejora del liderazgo pueden contribuir para mejorar la colaboración interprofesional en la APS y mejorar sus impactos en la atención a la salud.

## Introduction

Interprofessional collaboration is a process that involves professionals from different areas of health care, with coordination of different types of knowledge for the production of care ^1^. A study carried out in the primary health care (PHC) of the Brazilian Unified National Health System (SUS) identified two types of interprofessional collaboration: team collaboration and network and community collaboration ^2^.

One literature review gathers collaborative interprofessional practice and team work ^3^. Other review analyzes the four modes of interprofessional work: team work, interprofessional collaboration or collaborative interprofessional practice, coordination and networking ^4^. These authors note that team work is the core of interprofessional work, which is characterized by common objectives, shared identity, clarity of roles, interdependence and co-responsibility of team professionals. Interprofessional interaction and communication are recognized as constitutive domains of interprofessional work ^2^ ^,^ ^3^ ^,^ ^5^.

In this study, we adopted the concept of interprofessional collaboration that presents some key elements of the interprofessional work previously pointed out, but in a less systematized and less intense manner ^4^. Interprofessional collaboration also constitutes a strategy valued by universal health care models, which prioritize comprehensive care ^6^, an essential attribute of PHC models ^7^. By promoting interaction between different health care professionals, interprofessional collaboration contributes to reducing morbidity and hospitalizations ^8^, in addition to providing greater patient satisfaction and better health outcomes in PHC ^9^.

Managers have invested in the implementation of interprofessional collaboration in PHC teams in several countries, including Brazil, which since 1994 has implemented family health teams (FHT) ^10^ ^,^ ^11^. Each FHT brings together physicians, nurses, nursing assistants or technicians, and community health workers (CHW). As of 2022, more than 40,000 teams were responsible for providing health care to approximately 134 million people throughout the Brazilian territory ^11^.

The Brazilian National Primary Health Care Policy ^12^ (PNAB) emphasizes that the provision of comprehensive and continuous health care is the central purpose of the Family Health Strategy (FHS). In order to achieve this objective, FHS professionals must work in an interdisciplinary manner, combining knowledge from different disciplines and backgrounds, and seek to conduct interprofessional work in the aforementioned sense, for shared planning of health care and coordinated actions. It should be noted that interprofessional and interdisciplinary approaches are distinct, but complementary and have the potential to improve the quality of health care.

Several studies have analyzed interprofessional collaboration in PHC teams in high-income countries ^13^. In Brazil, there are still few studies focused on interprofessional collaboration in FHS. Matuda et al. ^5^ analyzed the perceptions of PHC professionals about interprofessional collaboration in the municipality of São Paulo and found the interaction between professional categories and production goals as themes related to interprofessional collaboration.

Araújo et al. ^14^ conducted a comparative case study and interviewed FHS professionals from the municipality of Sobral (Ceará State, Brazil) and from health care units in the municipality of Coimbra, Portugal, using an instrument developed based on the D’Amour model ^1^ to characterize interprofessional collaboration. However, these studies did not aim to analyze the implementation of interprofessional collaboration in FHS.

Over the past 10 years, implementation science has been widely used as a method to research barriers to the systematic adoption of evidence-based practices. This aims to improve the adoption and sustainability of these practices, directly impacting the quality and effectiveness of health care actions ^15^. At the same time, implementation science seeks to understand why adherence to certain innovations was successful in some places and unsuccessful in others ^16^ ^,^ ^17^.

Thus, we consider implementation science a valuable tool to trace barriers and facilitators for the introduction of interprofessional collaboration in PHC services. This can have practical implications for both local management and the workers involved.

This is one of the first studies to use an implementation science instrument to research interprofessional collaboration in PHC. The objective of this study is to analyze the perception of PHC workers about the factors that influence the adherence to interprofessional collaboration, from the perspective of implementation science.

## Method

This is a qualitative, descriptive and exploratory research, carried out in basic health units (UBS) in the Municipality of São Bernardo do Campo, São Paulo State, Brazil. São Bernardo do Campo has an estimated population of 844,483 inhabitants ^18^. Of these, 63.3% are registered in the 154 FHT ^12^.

### Selection of participants

Our qualitative sampling followed the assumptions described by Minayo ^19^: favor the choice of subjects with attributes that are intended to be learned in the research and consider a sufficient and diverse number of participants that enables recurrence of information and explanatory potential of the accounts. Thus, we invited to participate in the study: FHT workers (CHW, nurses, physicians, nursing technicians/assistants) and managers of three UBS in the municipality of São Bernardo do Campo from different regions, whose covered populations had different degrees of vulnerability, according to the São Paulo Social Vulnerability Index (IPVS) ^20^. We sought the diversity of subjects in relation to gender, age, profession, time working in the FHS, and association or not with family and community medicine residency or multiprofessional practice in family health, as we considered that these differences could enrich the collection and analysis of the workers’ perceptions.

### Data collection

As a technique for producing empirical data, we used the semi-structured interview, conducted using a plan composed of guiding questions, as it enables the modulation of the questions according to the verbalizations and reactions of the interviewees, aiming to understand the perceptions related to the experiences lived and reach the collective in the individual account, in the historical and social context ^19^.

We tested the plan in the pilot phase of the research to assess the clarity of the questions and terms used. To this end, we interviewed three workers from a UBS, whose transcribed material was not included in the analysis.

To select the UBS included in the study, the following procedure was adopted: (1) of the 31 UBS of the São Bernardo do Campo municipality with FHS for more than two years, 10 were excluded because the professionals had close contact with the main researcher (N.P.K.); (2) next, the remaining 21 UBS were grouped according to the IPVS for the registered population, which resulted in the classification according to IPVS as “very low”, “low-medium”, and “medium-high”; (3) we selected one UBS from each of these three categories. In the three UBS, four professionals per team and managers were invited to participate. In total, 15 PHC workers were interviewed. Interviews were scheduled in advance with each participant, being carried out in reserved settings within the UBS during working hours. The collection took place between September and October 2021. The interviews were recorded and transcribed and lasted an average of one hour and 20 minutes. There was no refusal to participate.

The interviews were conducted by a family and community physician (N.P.K.) who worked in the FHT and, at the moment, works in the management of PHC in the municipality of São Bernardo do Campo. Thus, we chose to exclude the UBS in which the researcher had worked and those directly related to her work as a manager, in an attempt to minimize the bias of responses by participants linked to the N.P.K. researcher.

The researcher ATCS supervised the study and analyzed the transcribed material, together with N.P.K. A.T.C.S. is a family and community physician, has worked in care and management in the city of São Paulo and conducts research in the area of PHC and health management. A.C.C.G.G. and M.P. work in the area of interprofessional education, with A.C.C.G.G. focusing on PHC, and M.P. on work management and interprofessional education. P.C.D.S. has a recognized work in health technology assessment.

#### Data collection instrument

To devise the interview plan, we used domains and constructs of the *Consolidated Framework for Implementation Research* (CFIR), one of the most frequently applied instruments in implementation research. The CFIR consists of 39 constructs organized into five domains, including intervention/innovation characteristics, outer setting, inner setting, individuals characteristics and process ^21^.

The constructs employed were selected according to the research objectives ^16^ and the intervention/innovation studied was interprofessional collaboration. Three CFIR domains were selected: (1) intervention characteristics, to understand the difficulties perceived in the implementation of interprofessional collaboration; (2) inner scenario, to describe factors related to the work setting in PHC that could influence the implementation and sustainability of interprofessional collaboration; and (3) individuals characteristics, to consider the interrelationships between professionals, and between them and the organization, given the relevance of social interaction and communication in interprofessional collaboration ^21^.

These three dimensions were selected because they are relevant to trace barriers and facilitators for the introduction of interprofessional collaboration in PHC services. However, we chose not to research the “outer setting” domain, which analyzes the interactions of the service with external organizations, external policies and incentives for the adoption of the intervention; and the “process” domain, which analyzes the implementation from planning to assessment ^21^, because interprofessional collaboration in PHC had already been introduced since the beginning of the FHS in the mid-1990s. These two domains could be subject to memory bias and would limit the selection of participants to those who had worked in the FHT since the initial implementation of the teams.

The interviews were conducted based on a plan consisting of the following topics: sociodemographic characteristics of the participants, aspects related to work in PHC and FHS, in addition to the characterization of the implementation of interprofessional collaboration, guided by the three CFIR domains mentioned above.

### Data analysis

The transcribed material was submitted to thematic content analysis, carried out by two researchers, who followed the predefined steps: pre-analysis, exploration of the material and treatment of the results and interpretation ^19^. Procedures such as condensation, coding and preparation of categories and themes were adopted according to deductive logic. NVivo 1.6.1 software (https://www.qsrinternational.com/nvivo/home) was used to support content analysis.

### Procedures to increase reliability

For adaptation and validation of the plan in the axis of the CFIR domains, we submitted the translation and adaptation for the appreciation of two researchers working in the area of implementation science. In addition, we requested feedback from participants on the transcribed material.

### Ethical procedures

The research was approved by the Research Ethics Committee of the University of São Paulo Teaching Hospital (opinion n. 4,406,541). Data confidentiality and privacy were guaranteed to participants. All participants signed the Informed Consent Form. As a guarantee of anonymity, the names of the participants were replaced by the letters M (physician), E (nurse), T (nursing technician or assistant), A (CHW) or G (manager).

## Results and discussion

Of the 15 participating FHS workers, most were women (73%) and aged between 22 and 59 years. The average time working in PHC in the municipality was eight years; and in the current FHT, 2.9 years. More information about the participants is shown in Box 1.

**Figure 1:**
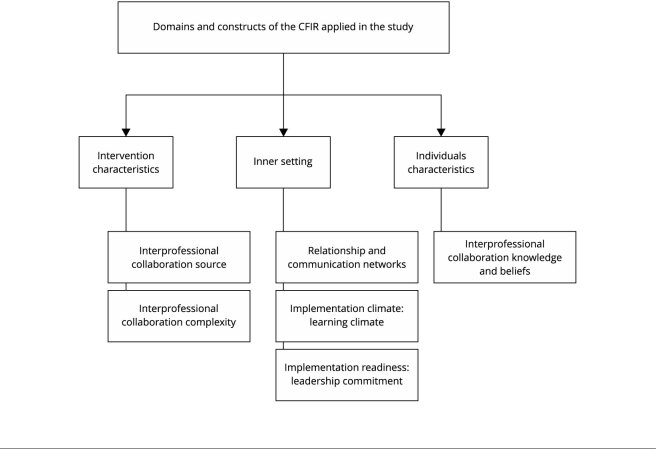
Domains and constructs of the *Consolidated Framework for Implementation Research* (CFIR) identified in the content analysis.

Below are the findings regarding the CFIR domains and their corresponding constructs, as shown in Figure 1.

**Box 1:** Respondent characteristics.

Participant code	Category	UBS	UBS area vulnerability degree according to IPVS (2010)	Age (years)	Biological sex
A1	CHW	1	Very low	58	Male
A2	CHW	2	Low to medium	29	Female
A3	CHW	3	Medium to high	45	Female
E1	Nurse	1	Very low	33	Female
E2	Nurse	2	Low to medium	38	Female
E3	Nurse	3	Medium to high	29	Male
G1	Manager	1	Very low	44	Female
G2	Manager	2	Low to medium	47	Female
G3	Manager	3	Medium to high	47	Female
M1	Physician	1	Very low	59	Male
M2	Physician	2	Low to medium	38	Male
M3	Physician	3	Medium to high	38	Female
T1	Nursing technician	1	Very low	40	Female
T2	Nursing assistant	2	Low to medium	55	Female
T3	Nursing technician	3	Medium to high	22	Female

CHW: community health worker; IPVS: São Paulo Social Vulnerability Index; UBS: basic health unit.

### Intervention Characteristics Domain

#### Construct - Interprofessional collaboration source 

Participants described interprofessional collaboration as a technology inherent to the competencies of PHC teams, conducted locally and directly associated with the integrated work of a team of professionals committed to the quality of the care provided to users.

“(...) *interprofessional collaboration is part of everyday routine in FHS... it is a work carried out by the team members, seeking the best for the patient. It is an exchange of information and experiences that lead to more comprehensive care. It integrates the vision of the CHW, the nurse and is complemented by the professionals of NASF* [Family Health Support Center]...” (M1).

Considering interprofessional collaboration as one of the axes of the work of FHTs focused on the quality of care is fundamental for PHC workers to establish spaces for the construction and articulation of knowledge and experiences of the various professional categories that make up the teams. Promoting continuing education on the subject and aligning concepts and impacts for care could contribute to improving implementation and sustainability, as proposed in the 2012 PNAB ^12^.

#### Construct - Interprofessional collaboration complexity 

According to the participants, the complexity of interprofessional collaboration arose from the recognition of the need to understand the role of each component of the team, to coordinate health care actions including the contribution of the various knowledge nuclei that compose the team, to build a common goal in each team, to deal with power asymmetry relations and to establish effective communication channels.

“*People are complex.* (...) *And then, this part, each one has to take a little bit of vanity aside and be more open to listening, you know?* (...) *the doctor, for example, is super open when she says something, some dressing conduct, because maybe it’s something that I have a little more experience than her. I think that when the team can be very aligned with a goal, when they can set expectations*” (E3).

“*I think* [the professionals know one another’s role] *very little and we would have to give conditions for this to happen* (...). *But it’s something that we can’t do. Why? Because the urgency of the service is overwhelming* (...). *So, you’re learning on the fly, you know?*” (G3).

“*And today I was discussing this case with the social worker and I said: ‘So what is your role in this story?’* (...). *Because sometimes we want so much from her and sometimes it's not all her responsibility, right?*” (M3).

The limited understanding of the role of each FHT worker, the communication difficulties to express ideas and the fear of not being accepted ^22^ can affect the quality of the care provided by the team, generate conflicts ^23^, duplicate health actions and underutilization of skills ^24^. Accordingly, the communication capacity of the leader, supervisor and manager is essential to highlight the duties of each worker, leading professionals to know their roles and those of colleagues ^24^, which leads to the reduction in power disputes, the integration of new roles in the team and interprofessional collaboration ^25^. The respondents described the importance of the leader’s communication skills for interprofessional collaboration, recognizing the knowledge and potential of each professional and exercising a shared and collaborative leadership.

“(...) *when he* [the team leader, nurse] *has something to say, he calls us, he asks, he says ‘What do you think? Look, this is happening’*. (...) *So, I think we feel valued, you know? And he, and he is always praising and talking about the importance of our work*” (A3).

Note that there is an interrelation between the points raised by the participants related to the “intervention complexity” construct (CFIR), the “shared vision/objectives” domains and the “leadership” of the interprofessional collaboration model of D’Amour et al. ^26^. Understanding the objectives shared by the team, focusing on the centrality of care, and having leaders who promote worker participation in decisions are key points, according to the referred model. Therefore, it is essential that the workers know the roles of each one and dedicate themselves to build health care, integrating the knowledge nuclei of the FHT.

### Inner Setting Domain

#### Construct - Relationship and communication networks 

This construct refers to the nature and quality of interaction and formal and informal relationship networks in an organization ^21^. According to the participants, the relevant aspects related to communication were: quality of the time reserved for formal communication between the team professionals and interactional aspects between the professionals.

#### a) Formal communication space

Although the spaces for formal communication in the FHT are part of the weekly schedule, lasting two hours, the participants’ accounts showed that the quality of the meeting is fundamentally important for interprofessional collaboration to be effective. The participants say that the lack or insufficiency of formal communication spaces constituted a barrier to interprofessional collaboration, impacting the quality of care, particularly for more complex cases followed-up by the team. The influence of interactions and communication in the implementation of interprofessional collaboration appeared more prominently in the accounts of FHT professionals who provide health care to populations in areas of greater vulnerability, which may be associated not only with the amount of demands, but with the complexity of cases from the perspective of intersectionality.

“*I think that, although we have a good dialogue* (...), *I feel that there is no time for us to be able to evaluate, to create therapeutic processes for patients* (...). *So, the demand is large...*” (E2).

Although the FHT meetings are aimed at identifying problems, making decisions for programming actions and evaluating activities ^27^, some authors report that PHC professionals mentioned problematic aspects related to the meetings, indicating that they were protocolary and with asymmetry of power between professionals, which made it difficult to express ideas and generated conflicts ^22^. Consistently, Carvalho et al. ^23^ found that such conflicts include disrespect stemming from asymmetric relations, the behavior of professionals and the lack of collaboration in the work. Savio et al. ^28^ described the use of messaging apps to service demands outside working hours, reducing the separation between personal life and work ^29^ and increasing stress and overload. It should be noted that face-to-face communication in in-person meetings has the advantage of enabling verbal and non-verbal exchanges ^28^ and making communication more effective. Thus, there should be discussion on how to improve the meeting and gathering spaces of the FHT. The better use of these spaces promotes a reduction in overload, exchanges between knowledge nuclei, development of health care plans and more effective interprofessional collaboration ^29^.

Communication in FHT should be conducted as a process of active and empathic listening ^30^, thereby contributing to reduce conflicts ^31^. Having times to improve communication, using specific techniques and non-violent communication, can improve relationships, increase empathy and trust, expand social support and have a direct impact on interprofessional collaboration ^32^. Interprofessional communication oriented to the health needs of users and the population of the territory is a sine qua non of team work and collaborative interprofessional practice ^33^.

#### b) Lack of professionals and turnover of FHT members

Lacking teams and high turnover of PHC professionals were considered limiting factors for the implementation and sustainability of interprofessional collaboration. These elements cause work overload, reduce the service time allocated to the team and can generate conflicts between professionals.

“*Make people relate, build relationships and that we have teams that can also have their time even to have their difficulties and then rebuild, because what we always see is that when it seems that everything is going well, then there is a doctor who leaves*...” (G3).

Longer coexistence in the same team can increase the maturation of the group ^14^. The high turnover of professionals makes it impossible to maintain integrated teams, which is fundamental for interprofessional collaboration, in addition to decreasing productivity and increasing costs due to the time spent on training and new hires ^34^ ^,^ ^35^.

#### Construct - Implementation climate: learning climate

This construct is related to the leaders’ role in promoting the valorization and contribution of team members in decision-making, with a safe space for reflection and evaluation ^21^. In this regard, the participants reported as relevant aspects related to the leaders’ commitment to interprofessional collaboration: feedback provided by leaders, promotion of autonomy, and participatory leadership.

#### a) Leaders’ feedback

Although feedback sessions have been considered strategies aimed at improving relationships at work, promoting learning and improving job satisfaction, the way feedback has been provided can be understood as a barrier to interprofessional collaboration, which can generate competition and demotivation.

“*So, when I worked with another nurse, I believe that the way he required the spreadsheets and everything else ended up generating* (...) *a kind of climate of competing* [with] *the other. ‘Oh, you-know-who made so many visits, you didn’t’*” (A2).

Some authors argue that feedback prioritizes reflection and recognition of strengths ^36^, provides social support, increasing motivation ^37^ and favoring interprofessional collaboration ^37^ ^,^ ^38^ ^,^ ^39^.

#### Construct - Implementation readiness: leadership commitment

#### a) Promotion of autonomy

The interviewees highlighted the importance of the manager stimulating autonomy and reducing dependence in decision-making. Some workers reported that having autonomy and feeling that the manager trusts the professional's work impacts the feeling of recognition and valorization.

“*And when they listen, when they are interested in the subject* (...). *Your voice is not a voice that is muffled* (...). *You have a voice, you know? What you said is important*” (A3).

“*At first, people thought it was strange. ‘Wait, are we supposed to meet among us to plan our receptionist schedule? Yeah, you’re the one who works as a receptionist. I can sit here with you, I’ll have a look’*” (G3).

Leaders who recognize and incorporate knowledge from the different groups ^40^ of professionals who make up PHC teams and value the ideas of team members ^41^ contribute to removing barriers to communication and favor interprofessional collaboration. Hjalmarson et al. ^37^, in a study involving PHC teams, observed that structures in which professionals are encouraged to act creatively and leaders encourage them to build proposed solutions to problems lead to greater interprofessional collaboration ^37^.

### Individuals Characteristics Domain

#### Construct - Interprofessional collaboration knowledge and beliefs

According to the respondents, sharing the same goals, objectives and responsibility for the health care of the population assigned to the team is a fundamental condition for interprofessional collaboration in the FHS.

“*That’s exactly it, that’s you*… *Everyone*… *having the same view to take care of the patient*” (G2).

Another common point observed in the participants’ reports was the relationship between team work and interaction and integration between professionals in the form of complementary work, joining forces, bonding, exchange of knowledge, information and experiences.

All team work characteristics presented in this study are present in the summary carried out by Reeves et al. ^42^ on elements that characterize teamwork: shared identity in the team, clarity of roles, common objectives, interdependence, integration, shared responsibility and activity to be performed ^42^. The results consist with the findings of a study with FHS professionals in Paraná State, which described the work of the FHT as a collective action, with integration and interdependence among professionals ^43^, as has been pointed out by other studies on the subject ^33^.

#### a) Perception about team members

Although all interviewed professionals are part of FHT, some participants did not include nursing technicians as components of the FHT. Often, nursing technicians are required to perform other tasks in the UBS rather than participating in the activities of the FHT itself. For example, precisely in the UBS where this professional was less mentioned as a team member, nursing technicians do not routinely participate in team meetings, they were on the shift for other activities in the UBS.

“*We have a doctor, a nurse, and a CHW. These are*... *this is the ‘core’, you know? But there is the NASF team that participates* (...)” (M1).

The non-description of the nursing technician as a member of the team was also pointed out in a study carried out in PHC in Paraná State ^44^, which highlighted the invisibility of the work of this professional category by the team itself.

Carvalho et al. ^23^ analyzed the perceptions of PHC workers and observed that asymmetric power relations among team members and lack of collaboration in work are related to conflicts in the team. The non-participation of nursing technicians in FHT meetings contributes to the perception described above and to perpetuate their invisibility in the team's work. Grando et al. ^22^ pointed out the challenges found in the FHT meetings so that this space is used as a possibility for collective building of health care. These authors observed that the meeting spaces are protocolary, centered on technical aspects and that professionals have difficulty expressing ideas and assuming a critical posture, which can lead them to be segregated by part of the group. This result indicates the limitations caused by the partial perception of the team members about their members, especially with regard to the lack of recognition of the role of some professions that compose the work team.

### Limitations and strengths of the study

The study presented as a limitation the fact that the main researcher is part of the central health care management of the municipality and has already worked in some UBS as a general medical practitioner and/or preceptor, having had previous social interaction with some interviewees. This may have increased the possibility of socially acceptable answers, despite the guarantee of information confidentiality and privacy. Another limitation to be highlighted concerns the selection of participants, as the study did not include the perception of oral health teams and NASF workers, which are part of PHC and contribute to the comprehensive care provided to the population in the FHS. In this study, we only sought the perception of workers from the minimum FHS teams (CHW, nursing technicians, nurses and physicians). We chose to include only FHT workers because we consider that they are present in most Brazilian municipalities and with greater continuity since their implementation, when compared to the oral health and NASF teams.

One of the strengths of the study is the use of an implementation science instrument to analyze interprofessional collaboration in the scope of PHC. Following the assumptions of implementation science studies, the composition of the research team included several knowledge nuclei ^15^ and intersecting areas for analysis and interpretation of results, as well as for the design of recommendations. Finally, it should be noted that the fact that the research was carried out in the COVID-19 pandemic period may contribute to improving interprofessional collaboration in PHC in future health crisis events.

## Conclusion

Our results can contribute to the development of actions by managers and health care workers to strengthen interprofessional collaboration in PHC. Actions to improve the implementation and sustainability of interprofessional collaboration in PHC include: (1) understanding one's role and the role of professionals from other areas in a work that seeks to produce interprofessional collaboration for the benefit of the health care of the covered population; (2) ensuring effective spaces for interaction and communication, formal and informal, especially in team meetings, to deal with the complexity of cases and demands according to the vulnerability of the FHT area; (3) recognizing and developing strategies to mitigate power asymmetries; (4) promoting permanent education focused on communication skills, active and empathic listening, ensuring a safe space for speaking and listening; (5) promoting permanent education of leaders and unit managers to provide feedback that includes work recognition, social support, improvement of skills and knowledge, reduction of conflicts and motivation for interprofessional collaboration.
